# Gender, GABAergic dysfunction and AD

**DOI:** 10.18632/aging.101672

**Published:** 2018-11-26

**Authors:** A. Alia, Steffen Roßner

**Affiliations:** 1 Institute of Medical Physics and Biophysics, University of Leipzig, D-04107 Leipzig, Germany; 2 Leiden Institute of Chemistry, Leiden University, 2333 CC Leiden, The Netherlands; 3 Paul Flechsig Institute for Brain Research, University of Leipzig, D-04103 Leipzig, Germany

**Keywords:** Alzheimer's disease, GABAergic dysfunction, sex difference

Alzheimer's disease (AD) is the most common cause of dementia and is characterized by a progressive decline of memory and other cognitive functions [1]. Emerging evidence suggests that AD disproportionately affects women in both occurrence and severity [1]. The molecular mechanism accounting for sex-related differences in AD remains unknown. Sex differences in amyloid β (Aβ) deposition as well as synaptic dysfunction in AD patients have been reported [2]. Gamma-aminobutyric acid (GABA) is the major inhibitory neurotransmitter in the central nervous system. Compelling evidence indicates that a disrupted default neuronal network underlies impaired memory and that alterations of GABAergic circuits may contribute to AD by disrupting the overall network function [3]. Whether GABAergic dysfunction is more severely affected in women than men remains to be established.

Our groups recently observed that progression of GABAergic dysfunction in an animal model of AD is clearly influenced by sex [4]. Using state-of-the-art high-resolution magic angle spinning NMR we systematically mapped the levels of GABA across several brain regions in male and female AD mice. Divergent changes in hippocampal and cortical GABA concentrations were observed, which were gender-specific. The GABA concentration in *prefrontal cortex* was more significantly affected in male as compared to female AD mice. In contrast, *hippocampal* GABA shows dynamic changes during Aβ pathology progression and its level was significantly elevated in old female as compared to old male AD mouse brain. To reveal the mechanism leading to high hippocampal GABA in female brain, we investigated the possible contribution of reactive astrocytes. Reactive astrocytosis is commonly observed in AD brain and much higher in female than male AD patients. While normal astrocytes do not contain much GABA, affected astrocytes around amyloid plaques become reactive and produce high amounts of GABA [5]. Till now, the role of reactive astrocytes has mainly been investigated in the context of inflammation or metabolic and structural support. Jo et al. [5] have recently reported the role of reactive astrocytes in producing GABA more aberrantly and abundantly in hippocampus, which was implicated in memory impairment in an AD mouse model. However, in those studies, the sex-specific differences in GABA production have not been evaluated. In our study, we found a significantly higher number of reactive astrocytes in hippocampus of female than male AD mouse brain, which correlated with higher number of Aβ plaques, especially in stratum *lacunosum moleculare* and in dentate gyrus of hippocampus [4]. The analysis of the enzymes involved in GABA synthesis demonstrated that glutamic acid decarboxylase (GAD) - which is essential for the production of GABA from glutamate in GABAergic neurons - displays low expression in hippocampus; especially in the areas surrounding Aβ plaques in both male and female AD mice. We then examined an alternative route of GABA production from putrescine via the monoamine oxidase-B (MAO-B) route, which predominantly exists in reactive astrocytes. The expression of MAO-B was clearly elevated GFAP-positive reactive astrocytes in hippocampus of female mice and was co-localized with putrescine immunoreactivity. These results indicate that an abnormal increase in tonic GABA release from reactive astrocytes in the hippocampus may be directly responsible for the memory impairment in AD.

The higher number of activated astrocytes and high amounts of hippocampal GABA in females may be associated with low estrogen levels in old females. Estrogen is known to act on hippocampal astrocytes of males and females via different mechanisms [1,6]. Additionally, estrogen-mediated neuroprotection in young female may partly be mediated by estrogen-astrocyte interaction [6]. Estrogen is also known to down-regulate the expression of MAO-B [7]. However, a decline in the estrogen level in old females may hamper the neuroprotective effect of astrocytes, which may be intervened by high production of GABA via MAO-B route. Abnormally high GABA release via the Best1 channels can then activate neuronal GABA receptors, which can strongly inhibit synaptic release, leading to impairment of synaptic plasticity and memory function. Taken together these findings signify that astrocytes play a significant role in synaptic dysfunction and memory impairment in AD. A sexual dimorphism of hippocampal astrocytes to produce GABA exists in AD and it can be at the root of marked sex disparities observed in AD incidence, manifestation, and prognosis.

The differential influence of sex on hippocampal GABA levels during AD pathogenesis offers unique challenges from the perspective of treating memory decline. While the suppression of GABA production or release from reactive astrocytes would bring about desirable therapeutic effects on memory impairment in AD, different therapeutic strategies may be required for improving hippocampus-dependent memory functions in male and female AD patients.
